# Protective Effect of Piplartine against LPS-Induced Sepsis through Attenuating the MAPKs/NF-κB Signaling Pathway and NLRP3 Inflammasome Activation

**DOI:** 10.3390/ph14060588

**Published:** 2021-06-18

**Authors:** Chi-Han Huang, Shu-Chi Wang, I-Chen Chen, Yi-Ting Chen, Po-Len Liu, Shih-Hua Fang, Shu-Pin Huang, Hsin-Chih Yeh, Ching-Chih Liu, Po-Yen Lee, Tzu-Chieh Lin, Wei-Chung Cheng, Chia-Cheng Su, Hsin-En Wu, Yuan-Ru Chen, Chia-Yang Li

**Affiliations:** 1 Graduate Institute of Medicine, College of Medicine, Kaohsiung Medical University, Kaohsiung 80708, Taiwan; cheryl60286@gmail.com (C.-H.H.); yljane.chen@gmail.com (I.-C.C.); yt0728@gmail.com (Y.-T.C.); retina.liu@gmail.com (C.-C.L.); maco69@gmail.com (P.-Y.L.); 990327kmuh@gmail.com (T.-C.L.); s940854@gmail.com (C.-C.S.); angxxie@gmail.com (H.-E.W.); yuannns90@gmail.com (Y.-R.C.); 2 Department of Medical Laboratory Science and Biotechnology, Kaohsiung Medical University, Kaohsiung 80708, Taiwan; shuchiwang@kmu.edu.tw; 3 Department of Pediatrics, Kaohsiung Medical University Hospital, Kaohsiung 80756, Taiwan; 4 Department of Pediatrics, School of Medicine, College of Medicine, Kaohsiung Medical University, Kaohsiung 80708, Taiwan; 5 Department of Pathology, Kaohsiung Medical University Hospital, Kaohsiung Medical University, Kaohsiung 80708, Taiwan; 6 Department of Pathology, Faculty of Medicine, College of Medicine, Kaohsiung Medical University, Kaohsiung 80708, Taiwan; 7 Department of Respiratory Therapy, College of Medicine, Kaohsiung Medical University, Kaohsiung 80708, Taiwan; kisa@kmu.edu.tw; 8 Institute of Athletics, National Taiwan University of Sport, Taichung 40404, Taiwan; shfang@ntupes.edu.tw; 9 Department of Urology, School of Medicine, College of Medicine, Kaohsiung Medical University, Kaohsiung 80708, Taiwan; shpihu73@gmail.com (S.-P.H.); patrick1201.tw@yahoo.com.tw (H.-C.Y.); 10 Department of Urology, Kaohsiung Medical University Hospital, Kaohsiung Medical University, Kaohsiung 80708, Taiwan; 11 Department of Urology, Kaohsiung Municipal Ta-Tung Hospital, Kaohsiung 80145, Taiwan; 12 Department of Ophthalmology, Chi Mei Medical Center, Tainan 71004, Taiwan; 13 Department of Ophthalmology, Kaohsiung Medical University Hospital, Kaohsiung Medical University, Kaohsiung 80708, Taiwan; 14 Division of Cardiology, Department of Internal Medicine, Kaohsiung Medical University, Kaohsiung 80708, Taiwan; 15 Graduate Institute of Biomedical Science, Research Center for Cancer Biology, China Medical University, Taichung 40403, Taiwan; Cwc0702@gmail.com; 16 Division of Urology, Department of Surgery, Chi-Mei Medical Center, Tainan 71004, Taiwan; 17 Department of Senior Citizen Service Management, Chia Nan University of Pharmacy and Science, Tainan 71710, Taiwan; 18 Department of Medical Research, Kaohsiung Medical University Hospital, Kaohsiung 80756, Taiwan

**Keywords:** piplartine, macrophage, inflammation, NLRP3 inflammasome, sepsis

## Abstract

Piplartine (or Piperlongumine) is a natural alkaloid isolated from *Piper longum* L., which has been proposed to exhibit various biological properties such as anti-inflammatory effects; however, the effect of piplartine on sepsis has not been examined. This study was performed to examine the anti-inflammatory activities of piplartine in vitro, ex vivo and in vivo using murine J774A.1 macrophage cell line, peritoneal macrophages, bone marrow-derived macrophages and an animal sepsis model. The results demonstrated that piplartine suppresses iNOS and COX-2 expression, reduces PGE_2_, TNF-α and IL-6 production, decreases the phosphorylation of MAPKs and NF-κB and attenuates NF-κB activity by LPS-activated macrophages. Piplartine also inhibits IL-1β production and suppresses NLRP3 inflammasome activation by LPS/ATP- and LPS/nigericin-activated macrophages. Moreover, piplartine reduces the production of nitric oxide (NO) and TNF-α, IL-6 and IL-1β, decreases LPS-induced tissue damage, attenuates infiltration of inflammatory cells and enhances the survival rate. Collectively, these results demonstrate piplartine exhibits anti-inflammatory activities in LPS-induced inflammation and sepsis and suggest that piplartine might have benefits for sepsis treatment.

## 1. Introduction

Sepsis, a life-threatening condition with 30–50% in-hospital mortality, has excessive inflammation in response to infection [1]. In the United States, 1.7 million adults experience inflammation annually, resulting in over 250,000 deaths [2,3]. Immune dysregulation is the core mechanism underlying the development of sepsis, which includes intense inflammatory response and cytokine storm [4]. Persistent increase in tumor necrosis factor (TNF)-α, interleukin (IL)-1β and IL-6 produced primarily by macrophages are associated with septic death [5]. During sepsis, monocytes are recruited and differentiate into inflammatory macrophages [6] that play a critical role in the maintenance of tissue homeostasis and contribute to the clearance of microorganisms and dying cells. In septic patients, the dysregulation of macrophage production has been demonstrated to be associated with adverse prognosis [7].

Lipopolysaccharide (LPS), the major outer surface membrane component of Gram-negative bacteria, is an important contributing factor to the pathogenesis of sepsis syndrome. LPS binds to toll-like receptor 4 (TLR4) and consequently activates the mitogen-activated protein kinase (MAPK) signaling pathway and induces an NF-κB-dependent inflammatory cascade, resulting in excessive production of proinflammatory mediators (nitric oxide (NO) and prostaglandin E2 (PGE_2_)) and cytokines (e.g., TNFα and IL-6) that are crucial in mediating the systemic inflammatory response during sepsis [8,9].

Inflammasomes are a group of multimeric protein complexes that are located mainly in immune cells such as macrophages. The activation of inflammasome results in the activation of caspase-1 proteolytic cleavage and consequently cleaves pro-IL-1β and pro-IL-18 into their active forms; they then, in turn, undergo pyroptosis induced by bacterial pathogens [10]. NLRP3 inflammasome has been regarded as a promising target for treating inflammation-associated diseases [11]. The NLRP3 inflammasome is activated in a two-step process, including both priming and activation steps. For the priming-step, it is induced through inflammatory stimuli such as LPS, a TLR4 agonist, which induces MAPK signaling (e.g., ERK, JNK and p38) and NF-κB-mediated NLRP3 and pro-IL-1β expression [12,13]. The activation step leads to assembling the NLRP3 inflammasome protein complex (consisting of NLRP3, ASC and pro-caspase-1) and activate caspase-1 cleavage, resulting in IL-1β and IL-18 maturation [14]. Targeting NLRP3 inflammasome has been considered as a novel therapeutic approach for treating sepsis [15,16].

Piplartine (or Piperlongumine), an alkaloid isolated from *Piper longum* L., has been demonstrated to exhibit various biological properties, including anti-inflammatory, anti-atherosclerotic and anti-tumor properties [17,18]. Piplartine suppresses the production of TNF-α and IL-6 and inhibits the activation of nuclear factor-κB (NF-κB) against proinflammatory responses and extracellular regulated kinases (ERK) 1/2 by LPS-activated human umbilical vein endothelial cells [10]; however, the anti-inflammatory effects of piplartine on LPS-activated macrophages and LPS-induced sepsis remain unclear. In this study, a series of assays were performed to examine the anti-inflammatory effects of piplartine in vitro, ex vivo and in vivo. The effect of piplartine on NLRP3 inflammasome activation was detected. The effect of piplartine on the LPS-induced sepsis mouse model was also determined.

## 2. Results

### 2.1. Piplartine Represses NO and PGE_2_ Production, Decreases COX-2 and iNOS Expression and Attenuates Proinflammatory Cytokine Secretion by LPS-Activated J774A.1 Cells

**Figure 1:**
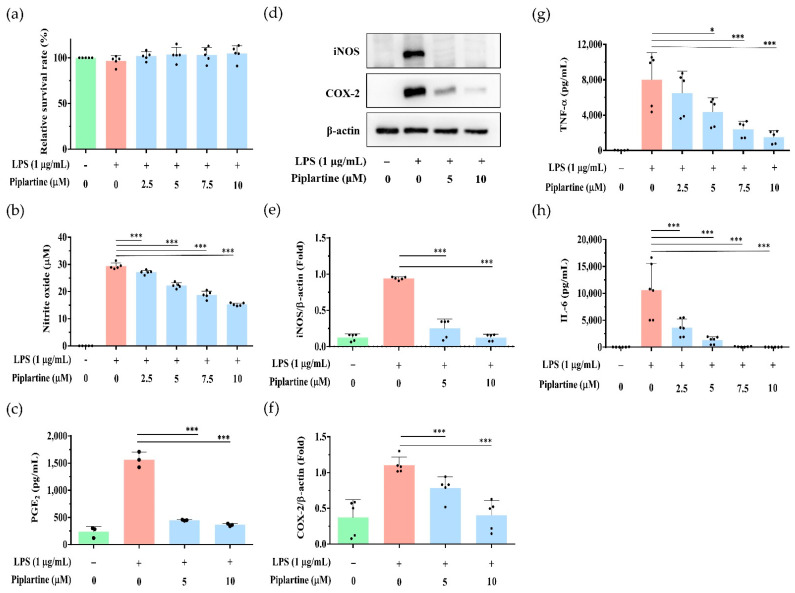
The effects of piplartine on the cell viability, NO and PGE_2_ production, iNOS and COX-2 expression and TNF-α and IL-6 secretion by LPS-activated J774.1 cells. Cells were pretreated with different concentrations (0–10 μM) of piplartine (1 h) and primed with 1 μg/mL LPS (24 h). (**a**) Cell viability was detected using MTT assay. (**b**) NO production was measured using Griess reagent assay. (**c**) The production of PGE_2_ was analyzed by ELISA. The expression of COX-2, iNOS and β-Actin (loading control) was determined by Western blotting. Representative images are shown in (**d**), and the quantified results from three independent experiments are shown in (**e**,**f**). The secretions of (**g**) TNF-α and (**h**) IL-6 were analyzed using ELISA. Data are presented as means ± SD and the significant differences indicated as * *p* < 0.05 and *** *p* < 0.001.

To elude the cytotoxic effects of piplartine, the effects of piplartine on the cell survival of J774A.1 cells were initially investigated. Cells were pretreated with different concentrations of piplartine (1 h) and primed with LPS (24 h). Cell viability was examined by the MTT assay. The experimental results indicated that the cell survival rate did not differ significantly when J774A.1 cells were treated with piplartine 0–10 μM (Figure 1a). To examine the effect of piplartine on LPS-induced NO and PGE_2_ production, J774A.1 cells were pretreated with piplartine in non-cytotoxic dosages (0–10 μM, 1 h) and primed with 1 μg/mL LPS (24 h). Our experimental results showed that piplartine significantly inhibited the production of NO and PGE_2_ by LPS-activated J774A.1 cells in a concentration-dependent manner (Figure 1b,c); additionally, we also examined the effects of piplartine on the expressions of iNOS and COX-2 by LPS-activated J774A.1 cells using western blot. As shown in Figure 1d–f, the experimental results indicated that piplartine significantly attenuated the expression of COX-2 and iNOS by LPS-activated J774A.1 cells as compared with LPS alone.

To further examine whether piplartine affected the secretion of proinflammatory cytokines by LPS-activated J774A.1 cells, cells were pretreated with different concentrations of piplartine (1 h) and primed with LPS (24 h). The expression levels of TNF-α and IL-6 in the cell culture supernatant were examined by ELISA. As shown in Figure 1 g,h, piplartine significantly attenuated the secretion of TNF-α and IL-6 by LPS-activated J774A.1 cells.

### 2.2. Piplartine Decreases the Phosphorylation of MAPKs, IκB and NF-κB by LPS-Activated J774A.1 Cells and Suppresses the Activation of NF-κB by LPS-Activated J-Blue Cells

**Figure 2:**
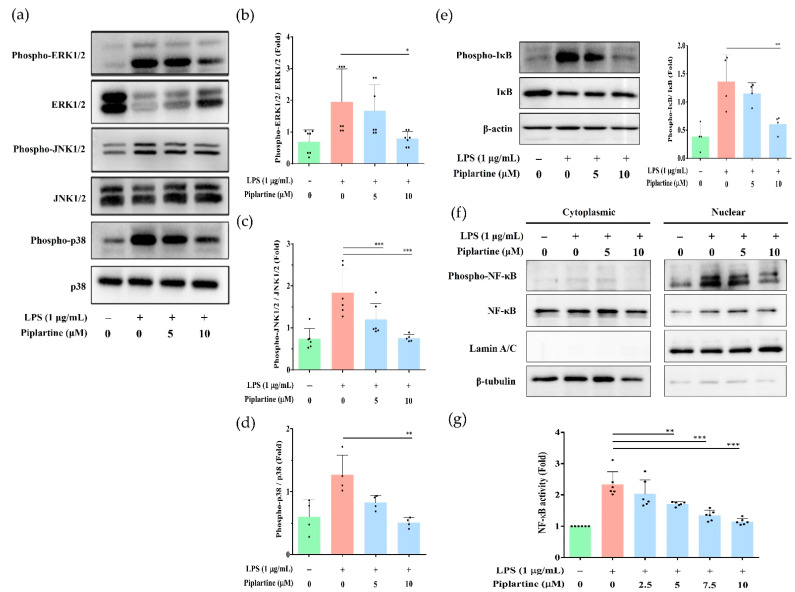
The effects of piplartine on both MAPKs and NF-κB signaling pathways in LPS-activated J774.1 cells. (**a**) Cells were pretreated with different concentrations (5 and 10 μM) of piplartine (1 h) and primed with 1 μg/mL LPS (2 h). Western blotting was performed to examine MAPK-associated protein expression. Representative results from one of three separate Western blotting experiments are shown, and the quantified results from three independent experiments are shown in (**b**–**d**). (**e**,**f**) Cells were pretreated with different concentrations (5 and 10 μM) of piplartine (1 h) and primed with 1 μg/mL LPS (30 min). Western blotting was executed to examine IκB protein expression in the cytoplasm and NF-κB protein expression between cytoplasm and nucleus. (**g**) J-blue cells were pretreated with different concentrations (0–10 μM) of piplartine (1 h) and primed with 1 μg/mL LPS (4 h). SEAP activity in the cell culture supernatant was examined. Data are presented as means ± SD and significant differences indicated as * *p* < 0.05, ** *p* < 0.01 and *** *p* < 0.001.

MAPKs play critical regulatory roles in the production of the proinflammatory cytokines and downstream signaling events that lead to inflammation [19,20]. In addition, NF-κB is a critical transcription factor in regulating inflammatory response development and progression [14]. To examine whether piplartine affected the MAPK signaling pathway activation by LPS-activated J774A.1 cells, cells were pretreated with different concentrations of piplartine (1 h) and primed with LPS (2 h). The expression of MAPK signaling-related proteins was detected using Western blotting. As shown in Figure 2a–d, the phosphorylation levels of JNK 1/2, p38 MAPK and ERK 1/2 were significantly elevated after LPS treatment, whereas piplartine significantly attenuated the phosphorylation of JNK 1/2, p38 MAPK and ERK 1/2 in LPS-activated J774A.1 cells. For detection of NF-κB expression and activation, the nuclear and cytoplasmic protein expression and promoter activity were examined by western blot and promotor reporter assay, respectively. As shown in Figure 2e, piplartine inhibited the phosphorylation of IκB in the cytoplasm. In addition, piplartine also suppressed the phosphorylation of NF-κB in the nuclear fraction compared with the LPS-alone group. Furthermore, the promoter activity of NF-κB was also examined using J-blue cells, a cell line derived from J774A.1 cells stably carrying the NF-κB reporter gene that induces secretion of embryonic alkaline phosphatase (SEAP) by NF-κB. The experimental results showed that piplartine significantly suppressed NF-κB activation by LPS-activated macrophages (Figure 2g).

### 2.3. Piplartine Decreases IL-1β Production and Represses NLRP3 Inflammasome Activation by LPS/ATP- and LPS/Nigericin-Activated J774A.1 Cells

**Figure 3:**
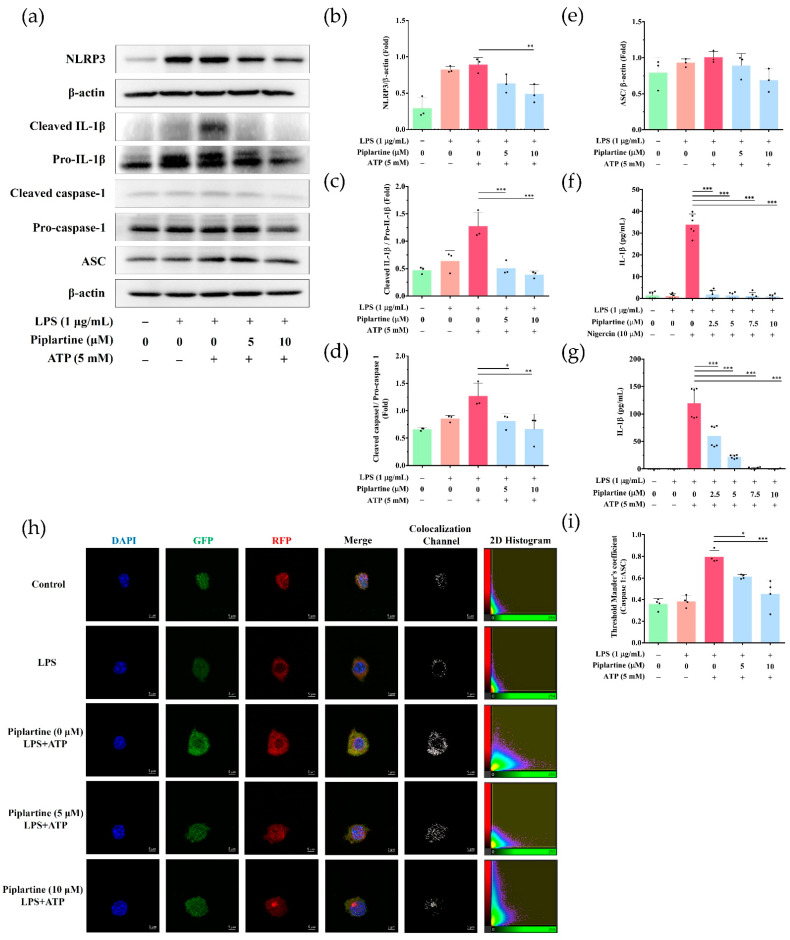
The effects of piplartine on NLRP3 inflammasome activation in macrophages. J774.1 cells were pretreated with different concentrations (0–10 μM) of piplartine (1 h), primed with 1 μg/mL LPS (5 h), and stimulated with 5 mM ATP (30 min). Inflammasome-associated protein expressions were detected using Western blotting, and β-Actin was used as a loading control. Representative images are shown in (**a**), and the quantified results from three independent experiments are shown in (**b**–**e**). (**f**,**g**) Cell culture supernatants were collected, and the levels of IL-1β were examined using ELISA. The colocalization of ASC (red) and caspase-1 (green), and DAPI in LPS/ATP-activated J774.1 cells was examined using confocal microscopy and Imaris software for confocal 3D image reconstruction. Representative images from three independent experiments are shown in (**h**). (**i**) The quantification of caspase-1 and ASC of colocalizated signals was analyzed using the threshold of the 2D histogram in panel (**h**) using Mander’s coefficient. Significant differences are indicated as * *p* < 0.05, ** *p* < 0.01 and *** *p* < 0.001.

NLRP3 inflammasome activation plays a critical role in regulating IL-1β production, which is an important regulator in modulating inflammatory responses [21]. To examine whether piplartine affected NLRP3 inflammasome activation, J774A.1 cells were pretreated with different concentrations of piplartine (1 h), primed with LPS (5 h), and stimulated with ATP (30 min) or nigericin (30 min). The expression levels of IL-1β in the cell culture supernatant were detected using ELISA, whereas the expression of inflammasome-related proteins was examined using Western blotting. The experimental results showed that piplartine inhibited NLRP3, pro-caspase-1, cleaved caspase-1, pro-IL-1β and cleaved IL-1β expressions in LPS/ATP-activated J774A.1 cells, but had no effect on ASC expression (Figure 3a–e). In addition, piplartine also significantly suppressed IL-1β secretion by LPS/nigericin and LPS/ATP-activated J774A.1 cells (Figure 3f,g). Moreover, the colocalization of NLRP3 inflammasome components (ASC and caspase-1) was also examined as this is considered a marker for inflammasome assembly [22]. Experimental results indicated that piplartine decreased the colocalization of caspase-1 and ASC in LPS/ATP-activated J774A.1 cells (Figure 3h,i).

### 2.4. Piplartine Represses NO and Proinflammatory Cytokine Productions by LPS-Activated Murine Peritoneal and Bone Marrow-Derived Macrophages

**Figure 4:**
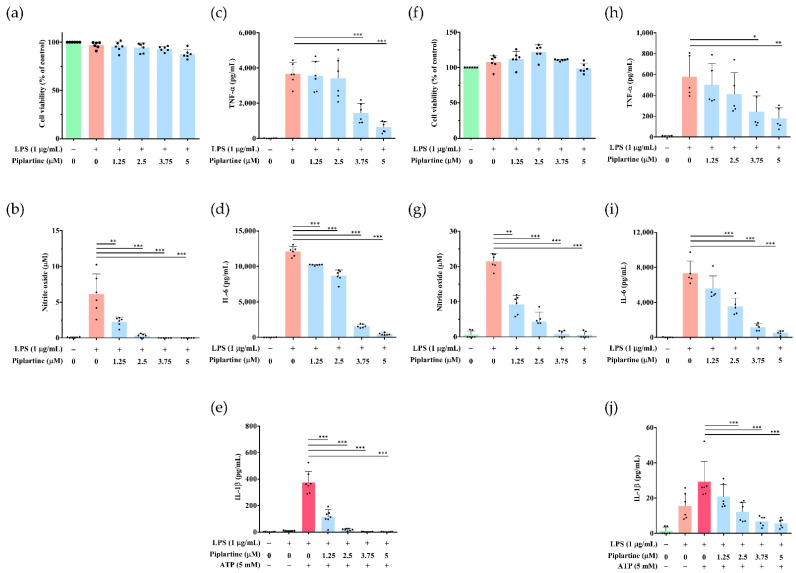
The anti-inflammatory effects of piplartine on murine peritoneal and bone marrow-derived macrophages. (**a**,**f**) Cells were pretreated with different concentrations (0–5 μM) of piplartine (1 h) and then treated with 1 μg/mL LPS (24 h). Cell viability was analyzed by MTT assay. (**b**,**g**) The production of NO was determined by Griess reagent assay. The levels of (**c**,**h**) TNF-α and (**d**,**i**) IL-6 were detected using ELISA. (**e**,**j**) Cells were pretreated with different concentrations (0–5 μM) of piplartine (1 h), primed with 1 μg/mL LPS (5 h), and stimulated with 5 mM ATP (30 min). Cell culture supernatants were harvested, and levels of IL-1β were examined using ELISA. Significant differences are indicated as * *p* < 0.05, ** *p* < 0.01 and *** *p* < 0.001.

The above experimental results indicated that piplartine significantly inhibited LPS-induced inflammatory mediator (NO and PGE_2_) and proinflammatory cytokine (TNF-α, IL-6 and IL-1β) secretion by J774A.1 cells, a murine macrophage cell line. The anti-inflammatory activities of piplartine were further verified using primary macrophages, murine peritoneal and bone marrow-derived macrophages. Thioglycolate-elicited peritoneal macrophages were incubated with different concentrations (0–5 μM) of piplartine (1 h) and primed with 1 μg/mL LPS (24 h). As shown in Figure 4a, cell survival rate showed no significant difference when cells were treated with 0–5 μΜ piplartine. In addition, piplartine significantly inhibited NO, TNF-α and IL-6 productions by LPS-activated murine peritoneal macrophages (Figure 4b–d), and it was also found that piplartine significantly inhibited the production of IL-1β by LPS/ATP-activated murine peritoneal macrophages (Figure 4e). On the other hand, the experimental results indicated that there was no cytotoxic effect in murine bone marrow-derived macrophages when cells were treated with ≤5 μM piplartine (Figure 4f). As shown in Figure 4g–i, piplartine significantly suppressed IL-6 and TNF-α production by LPS-activated murine bone marrow-derived macrophages and inhibited IL-1β production by LPS/ATP-activated murine bone marrow-derived macrophages (Figure 4j).

### 2.5. Piplartine Exhibits Protective Effects against LPS-Induced Tissue Damages and Increases the Survival Rate of LPS-Challenged Mice

**Figure 5:**
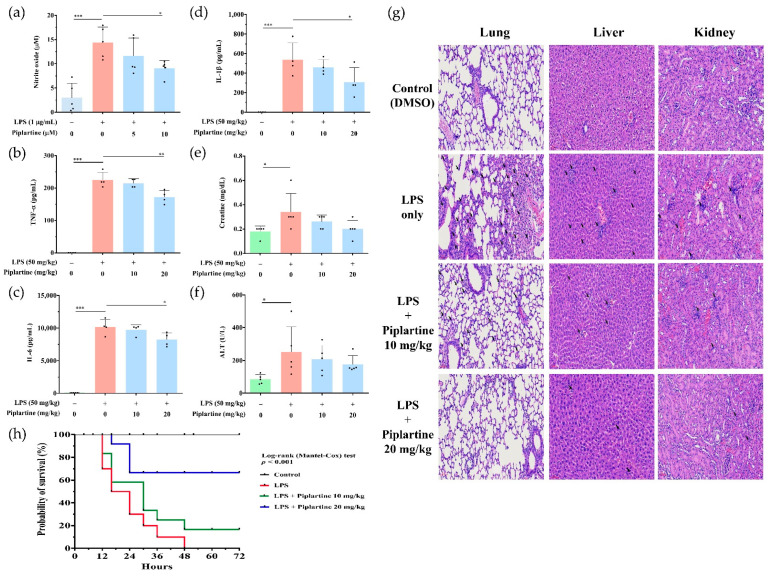
Effects of piplartine on serum NO, TNF-α, IL-6, IL-1β levels, liver and kidney damage markers and mortality in LPS-challenged mice. Mice were intraperitoneally injected with piplartine (10 or 20 mg/kg) or DMSO (control) for 1 h before 50 mg/kg LPS intraperitoneal injection, and the blood samples were collected 4 h after LPS injection. (**a**) The level of NO in the serum was measured by Griess reagent assay. The levels of (**b**) TNF-α, (**c**) IL-6 and (**d**) IL-1β in the serum were measured by ELISA. (**e**,**f**) The levels of CRE and ALT were measured (n = 4). Statistical significance was assessed by one-way ANOVA followed by Tukey post-hoc test and represented as follows: * *p* < 0.05, ** *p* < 0.01 and *** *p* < 0.001. (**g**) Tissues of lung, liver and kidney were harvested after LPS injection 4 h and stained by H&E-staining kit (200x magnification). The damaged sites and infiltration of inflammatory cells are indicated by black arrows. The figure is representative data from three independent experiments. (**h**) The survival rate was monitored at different intervals (*n* = 10). Statistical significance was analyzed using the log-rank test and represented as follows: * *p* < 0.05 vs. DMSO.

Murine endotoxemia model through LPS injection is a commonly used animal model for sepsis study, which presents overreactive inflammatory responses and acute tissue injuries, including lung, liver and kidney [23], so the anti-inflammatory effects of piplartine were further investigated using LPS-challenged mice. Mice were intraperitoneally injected with piplartine (1 h) and then intraperitoneally injected with LPS to induce experimental sepsis. After the administration of LPS for 4 h, blood was collected, and the levels of NO, TNF-α, IL-6, IL-1β, creatinine and ALT were analyzed, and lung, liver and kidney tissues were harvested for HE staining. As shown in Figure 5a–f, piplartine significantly suppressed the production of NO, TNF-α, IL-6 and IL-1β and moderately attenuated the levels of creatinine (CRE) and alanine aminotransferase (ALT) in LPS-challenged mice. As shown in Figure 5g, lung injury (increase in alveolar wall thickness and increase in inflammatory cell infiltration), liver injury (increase in inflammatory cell infiltration) and kidney injury (increase in inflammatory cell infiltration) were found in the LPS-challenged group. Notably, the administration of piplartine revealed a protective effect by attenuating lung, liver and kidney injuries (Figure 5g). During sepsis, the overwhelming production of proinflammatory cytokines and mediators leads to an increase in tissue damage or lethality. Our results showed that piplartine inhibited proinflammatory cytokine and mediator productions (Figure 5a–f) and protected tissue damages (Figure 5g) in LPS-challenged mice. In addition, whether piplartine possessed protective effects on LPS-induced septic shock was further examined. Mice were intraperitoneally injected with 10 or 20 mg/kg piplartine (1 h) and then intraperitoneally injected with 50 mg/kg LPS. The survival rate was monitored for 5 days. The experimental results indicated that piplartine significantly increased the survival rate compared to the LPS-alone group (Figure 5h).

## 3. Discussion

*Piper longum* L. is an important and common ingredient in Ayurvedic medicine, which has various biological activities, such as cardiovascular activities, anti-inflammatory activity, anti-tumor activity [24,25]. Piplartine is one of the main bioactive components found in *Piper longum* L. Recent studies indicated that piplartine has anti-cancer activities in several types of cancers, including glioblastoma [17], colon cancer [26], skin cancer [27], leukemia [28], hepatocellular carcinoma [29]. In addition, piplartine also exhibits antiparasitic activity [30,31,32,33]; however, the effect of piplartine on immunoregulation has not been well investigated.

LPS, a TLR4 agonist, is commonly used for triggering inflammatory responses in macrophages by the production of inflammatory mediators (PGE_2_, iNOS and COX-2) and proinflammatory cytokines (TNF-α and IL-6). For both in vitro and ex vivo experiments, the anti-inflammatory effects of piplartine were tested using three types of macrophages, murine J774A.1 macrophage cell line, peritoneal and bone marrow-derived macrophages. Our results pointed out that LPS significantly increased PGE_2_, NO, IL-6 and TNF-α secretions and promoted iNOS and COX-2 expressions by macrophages, while piplartine treatment exhibited anti-inflammatory effects by repressing PGE_2_, NO, IL-6 and TNF-α secretions and attenuating iNOS and COX-2 expressions by LPS-activated macrophages. A previous study showed that piplartine suppresses the production of TNF-α or IL-6 and NF-κB or extracellular regulated kinases (ERK) 1/2 by LPS-activated human umbilical vein endothelial cells [34]. Both MAPK- and NF-κB-signaling pathways play important roles in regulating inflammatory responses and leading to proinflammatory cytokine production when activated [35]. As our results show, piplartine significantly inhibited ERK, JNK and p38 phosphorylation by LPS-activated J774A.1 cells. In addition, piplartine also attenuated the activity of NF-κB by LPS-activated J-blue cells and suppressed the phosphorylation of IκB and NF-κB by LPS-activated J774A.1 cells. Collectively, these results suggest that piplartine suppresses the secretion of NO, PGE_2_, IL-6 and TNF-α by inhibiting both MAPK- and NF-κB-signaling pathways.

The NLRP3 inflammasome serves as a major component of innate immunity and is regarded as a potential therapeutic target for treating inflammation-associated diseases, including sepsis [15,16,36]. Pyroptosis is mainly mediated by inflammatory caspases (caspase-1/4/5/11) that cleave and activate the pyroptotic substrate gasdermin D (GSDMD) that promotes the physical rupture of pyroptotic cells, resulting in the release of the proinflammatory cytokines IL-1b and IL-18, alarmins and endogenous danger-associated molecular proteins [37].

Our results indicated that piplartine suppressed NLRP3, cleaved caspase-1, pro-IL-1β and cleaved IL-1β expressions, and repressed IL-1β secretion as well as attenuating caspase-1 and ASC colocalization in LPS/ATP-activated J774A.1 cells. Moreover, piplartine also inhibited IL-1β secretion by LPS/nigericin-activated J774A.1 cells. These results indicate that piplartine attenuates IL-1β production by attenuating NLRP3 inflammasome activation. To our knowledge, this is the first study to demonstrate the inhibitory effect of piplartine on NLRP3 inflammasome activation by macrophages.

Several murine models of sepsis have been used for pathological studies and drug development, including the endotoxemia model, bacterial injection model, cecal ligation and puncture, colon ascendens stent peritonitis [38]. Murine endotoxemia model through LPS injection is commonly used for the experimental studies of sepsis with several advantages, such as well-controlled LPS dosage and short handling time, and have been employed in an effort to recapitulate key features of severe human sepsis [38,39]. Our experimental results indicated that treatment with piplartine results in the reduction of LPS-induced septic mortality; additionally, piplartine also significantly suppressed the production of NO, TNF-α, IL-6 and IL-1β. These results suggest that piplartine has protective effects from the immune over-activated inflammatory reaction by reducing the production of NO, proinflammatory cytokines and inhibiting activation of NLRP3 inflammasome.

## 4. Materials and Methods

### 4.1. Cell Culture

The murine macrophage cell line (J774A.1 cell) and murine fibroblast cell line L-929 were purchased from Bioresource Collection and Research Center (Hsinchu, Taiwan) and maintained in Dulbecco’s Modified Eagle’s Medium (DMEM) and Roswell Park Memorial Institute (RPMI)-1640 media, respectively. All cell culture media were supplied with 10% (*v/v*) heat-inactivated fetal bovine serum (Life Technologies, Grand Island, NY, USA), 100 U/mL penicillin and 100 U/mL streptomycin. Cells were cultured at a humidified atmosphere of 5% CO_2_ at 37 °C in a humidified atmosphere of 5% CO_2_ and passaged every 2–3 days to maintain growth.

### 4.2. Preparation of Peritoneal and Bone Marrow-Derived Macrophages

Peritoneal macrophages were isolated from the C57BL/6 mice as described previously [12,14,21]. Briefly, mice were injected intraperitoneally with 1 mL sterile 3% thioglycollate medium (purchased from Sigma Aldrich, St. Louis, MO, USA) for 4 days. The peritoneal exudate cells were aseptically isolated via peritoneal lavage with cold PBS. Cells were maintained in RPMI-1640 medium at a density of 1 × 10^6^ cells/mL and incubated for 4 h at 37 °C. Afterward, non-adherent cells were removed by gently washing twice with warm PBS, and adherent peritoneal macrophages were used for following piplartine and LPS treatments. For the bone marrow-derived macrophage differentiation, bone marrow cells were harvested from tibias and femurs of C57BL/6 mice as described previously [20] and cultured in RPMI medium, and 15% L929 conditioned medium for 7 days. Adherent cells were used as macrophages [21].

### 4.3. Animals

C57BL/6 female mice (6–8 weeks) were purchased from the National Lab Animal Center (Taipei, Taiwan). All animal care and experimental procedures were performed under previously approved protocols by the Institutional Animal Care and Use Committee on the Ethics of Animal Experiments of Kaohsiung Medical University (Permit Number: 108071, approval date: 1 September 2019~31 August 2022).

### 4.4. Cytotoxicity Assay

Cells were seeded in 96-well plates (1 × 10^5^ cells/mL) overnight. Afterward, cells were pretreated with different concentrations (0–10 μΜ) of the piplartine (obtained from ChemFaces, Wuhan, Hunan, China) for 1 h, and then primed with 1 μg/mL LPS (from *E. coli.* O111:B4, purchased from Sigma Aldrich) for 24 h. MTT (5 mg/mL, purchased from Sigma Aldrich) was added to each well, and the plates were incubated at 37 °C for 4 h. The formazan crystals were lysed with 0.04 M HCl/isopropanol. The absorbance was measured by a microplate reader at 570 nm (BioTek Instruments, Winooski, VT, USA).

### 4.5. NO Assay

NO production was examined by detecting nitrite (its stable end product) by the Griess reagent according to the manufacturer’s protocol (Sigma Aldrich). A total of 1 × 10^5^ cells were seeded in 96-well plates and then incubated at 37 °C overnight. Afterward, cells were pretreated with different concentrations (0–10 μM) of piplartine (1 h) and primed with 1 μg/mL LPS (24 h). A total of 100 μL cell culture supernatant was collected, mixed with 100 μL Griess reagent, and incubated at room temperature for 10 min. The absorbance at 540 nm was measured using a microplate reader (BioTek Instruments), and the quantity of nitrite was calculated from a sodium nitrite standard curve.

### 4.6. Measurement of Cytokine Secretion

A total of 1 × 10^5^ cells were seeded in 96-well plates and incubated overnight. Afterward, cells were pretreated with different concentrations (0–10 μM) of piplartine (1 h) and primed with 1 μg/mL LPS (24 h). Cell culture supernatant was collected, and the levels of TNF-α, IL-6 and PGE_2_ were measured using ELISA according to the manufacturer’s protocols (Thermo Scientific, Waltham, MA, USA). For the determination of the IL-1β, cells were pretreated with different concentrations (0–10 μM) of piplartine (1 h), primed with 1 μg/mL LPS (5 h), and stimulated with 5 mM ATP (30 min) or 10 μM nigericin (30 min). Cell culture supernatants were harvested, and the levels of IL-1β were detected using ELISA (Thermo Scientific).

### 4.7. Western Blotting Analysis

Cells were washed three times with PBS and then lysed using RIPA buffer containing protease inhibitors and phosphatase inhibitors (Sigma Aldrich). For the nuclear and cytoplasmic protein fractions, the protein extraction was performed by nuclear extraction-isolation kit according to the manufacturer’s protocol (Fivephoton Biochemicals, San Diego, CA, USA). The concentration of protein lysates was measured by BCA reagent (Thermo Scientific). Equal amounts of protein were separated by SDS-PAGE and then transferred to PVDF membranes. The membrane was incubated in blocking buffer for 1 h at room temperature and then probed with primary antibodies (Supplemental Table S1) at 4 °C (1:1000) overnight. Blots were washed three times with TPBS containing 0.05% Tween-20 and incubated with horseradish peroxidase (HRP)- conjugated secondary antibody (1:5000) (Santa Cruz, Santa Cruz, CA, USA) for 1 h at room temperature, followed by chemiluminescent detection by a Bio-Rad ChemiDoc XRS^+^ system (Bio-Rad Laboratories, Inc., Hercules, CA, USA).

### 4.8. NF-κB Promoter Activity Assay

J-blue cells are a cell line derived from J774A.1 cells stably carrying the NF-κB reporter gene that induces SEAP secretion by NF-κB, which was maintained in DMEM medium supplemented with Zeocin (200 μg/mL) (InvivoGen, San Diego, CA, USA). A total of 1 × 10^5^ cells were seeded in 96-well plates and grown overnight in a 5% CO_2_ incubator at 37 °C. Afterward, cells were pretreated with different concentrations (0–10 μM) of piplartine (1 h) and then treated with LPS (1 μg/mL) (4 h) [14]. Cell supernatant was harvested and mixed with QUANTI-blue medium (20 μL cell culture supernatant to 200 μL QUANTI-blue medium, purchased from InvivoGen) in 96-well plates and incubated at 37 °C for 45 min. SEAP activity was then assessed by measuring the optical density at 655 nm using an ELISA reader.

### 4.9. Immunofluorescence Staining

The procedure of immunofluorescence staining was following our previous study [40]. Primary antibodies against ASC and caspase-1 were shown in Supplemental Table S1. For quantitation of fluorescence intensity, multiple micro photos were acquired randomly under a 63x objective using a confocal laser microscope (Leica, Exton, PA, USA) and analyzed using Imaris 8 Image Analysis Software (Oxford Instruments, Oxford, UK).

### 4.10. Endotoxemia Animal Model and Histological and Biochemistry Analysis

Female C57BL/6 mice (6–8 weeks) were injected intraperitoneally with DMSO (control group) or piplartine (10 or 20 mg/kg) 1 h after intraperitoneal injection of LPS (50 mg/kg). Blood samples were harvested after LPS injection (4 h) for NO, TNF-α, IL-6, IL-1β, CRE and ALT examination, and then sacrificed for tissue collection (lung, liver and kidney). Tissues were fixed using 4% formaldehyde, embedded with paraffin, and stained by hematoxylin and eosin (H&E) staining. The level of NO and proinflammatory cytokines were analyzed by Griess reagent assay and ELISA, respectively. The levels of ALT and CRE were detected by a Fuji Dri-Chem 3500i biochemistry analyzer (Fujifilm Ltd., Japan). For evaluating the survival rate, mice were monitored for 5 days at different intervals after piplartine and LPS administration and the log-rank statistical method was applied.

### 4.11. Statistical Analysis

The experimental results are presented as mean ± standard deviation (SD). All data shown are from three independent experiments. Statistical analysis was performed by a one-way ANOVA followed by Tukey post-hoc test using GraphPad Prism 6 (San Diego, CA, USA). The significant difference between the groups was defined as *p < 0.05; **p < 0.01.

## 5. Conclusions

**Figure 6:**
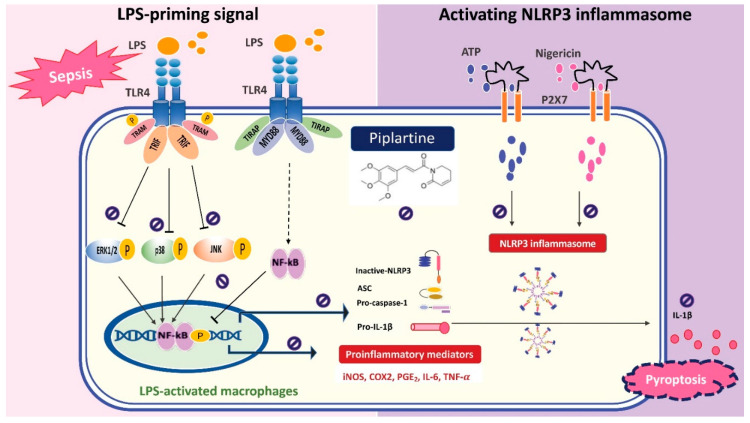
Piplartine suppresses the production of proinflammatory mediators and cytokines through inhibiting MAPKs/NF-κB signaling pathways and NLRP3 inflammasome activation by LPS-activated macrophages and protects LPS-induced septic shock.

Our experimental results demonstrated that piplartine suppresses the production of proinflammatory mediators and cytokines through inhibiting MAPKs/NF-κB signaling pathway and NLRP3 inflammasome activation by LPS-activated macrophages and protects LPS-induced septic shock, suggesting that piplartine might have benefits for treating sepsis (Figure 6). Therefore, piplartine could be a potential agent to combat bacterial sepsis.

## Data Availability

Data is contained within the article and supplementary material.

## Supplementary Materials

The following are available online at https://www.mdpi.com/article/10.3390/ph14060588/s1, Table S1: List of primary antibodies used in this study.
