# Supplementary material for: Protective Effect of Piplartine against LPS-Induced Sepsis through Attenuating the MAPKs/NF-κB Signaling Pathway and NLRP3 Inflammasome Activation
Source: Pharmaceuticals (Basel). 2021 Jun 18;14(6):588. doi: 10.3390/ph14060588 (PMC8234963; doi:10.3390/ph14060588)
Supplement: Supplementary file 1 [file pharmaceuticals-14-00588-s001.zip › pharmaceuticals-1240855-supplementary.pdf]

Supplemental Table S1. List of primary antibodies used in this study.

Antibodies	Catalog number	Company
Nitric oxide synthase (iNOS)	sc-651	Santa Cruz Biotechnology (Santa Cruz, CA)
Cyclooxygenase 2 (COX-2)	sc-166475	Santa Cruz Biotechnology
ASC	sc-22514-R	Santa Cruz Biotechnology
Caspase-1 p10	sc-514	Santa Cruz Biotechnology
IL-1β	sc-7884	Santa Cruz Biotechnology
β-tubulin	sc-5274	Santa Cruz Biotechnology
Phospho-ERK1/2	CST#4370	Cell Signaling (Farmingdale, NY)
ERK1/2	CST#4695	Cell Signaling
Phospho-JNK 1/2	CST#9255	Cell Signaling
JNK 1/2	CST#9258	Cell Signaling
Phospho-p38 MAPK	CST#4511	Cell Signaling
p38 MAPK	CST#8690	Cell Signaling
Phospho-NF-κB	CST#3033	Cell Signaling
NF-κB p65	CST#8284	Cell Signaling
Phospho-IκB	CST#2859	Cell Signaling
IκB	CST#4814	Cell Signaling
NLRP3	CST#15101	Cell Signaling
Cleaved caspase-1	CST#67314	Cell Signaling
Cleaved IL-1β	CST#52718	Cell Signaling
β-actin	GTX629630	GeneTex (Irvine, CA)
Lamin A+C	GTX101127	GeneTex

Supplemental material: Western blot raw images.

Figure.S1

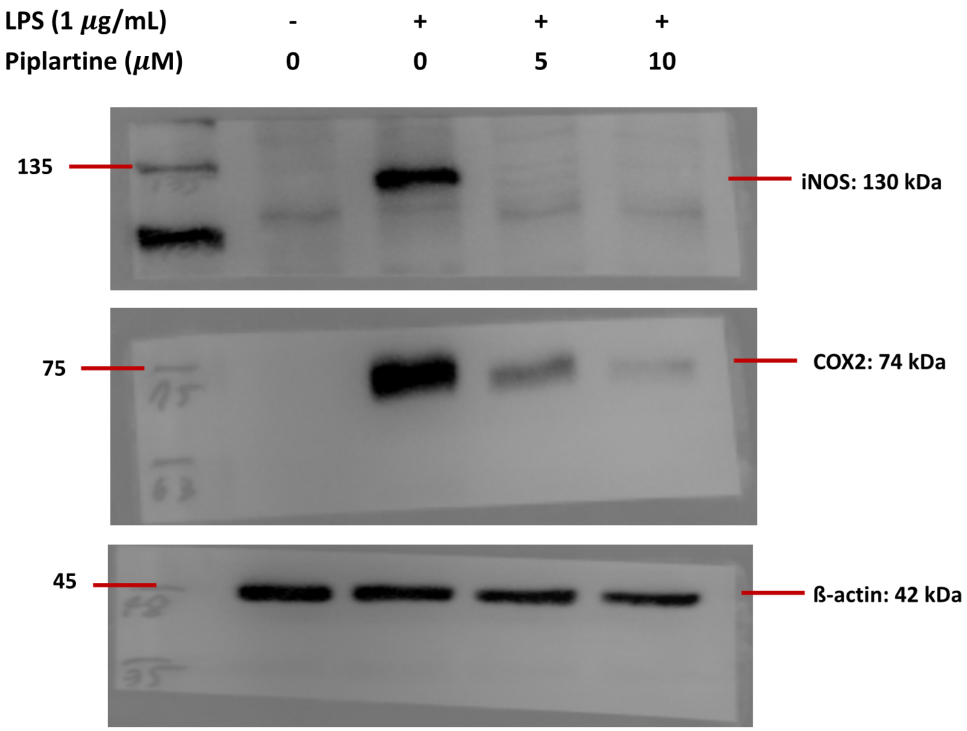

Figure.S2

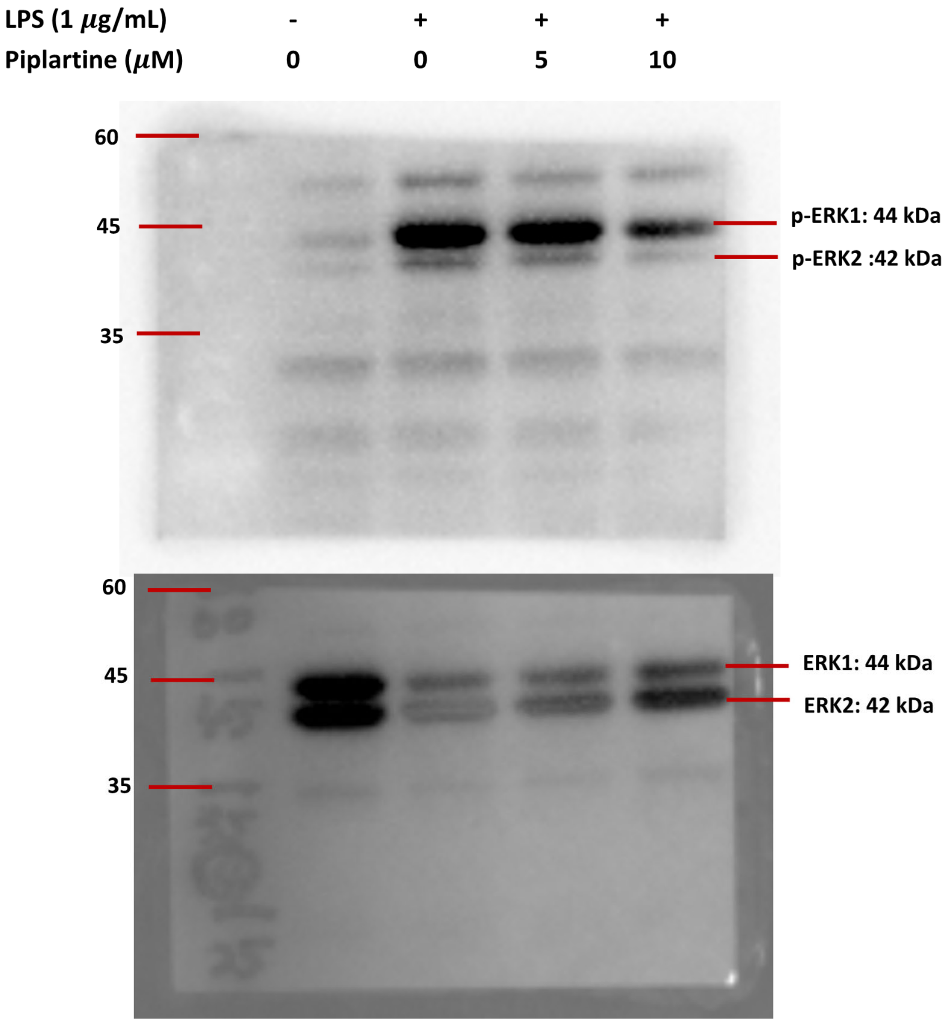
LPS (1 μg/mL) - + + +
Piplartine (μM) 0 0 5 10
60
45
35
p-ERK1: 44 kDa
p-ERK2 :42 kDa
60
45
35
ERK1: 44 kDa
ERK2: 42 kDa

Figure.S2

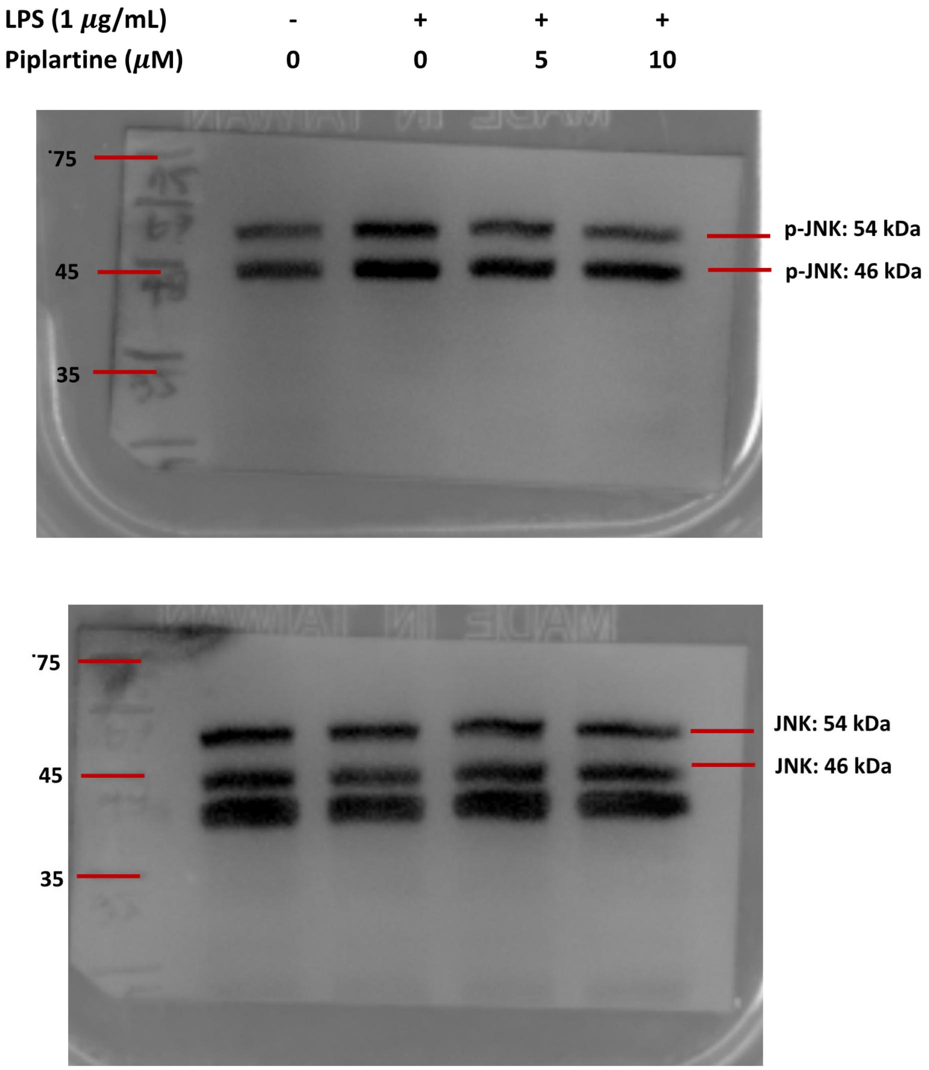

LPS (1 μg/mL) - + + +
Piplartine (μM) 0 0 5 10
75
45
35
p-JNK: 54 kDa
p-JNK: 46 kDa
75
45
35
JNK: 54 kDa
JNK: 46 kDa

Figure.S2

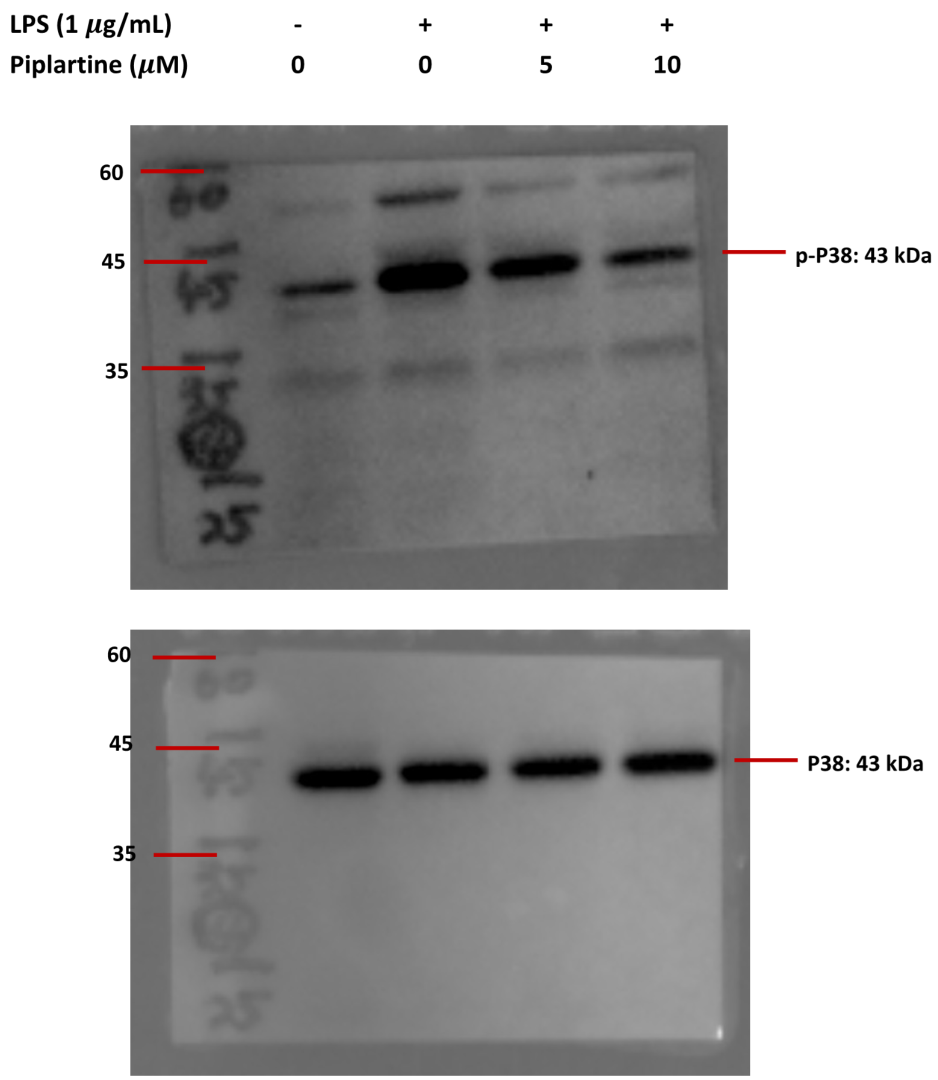
LPS (1 μg/mL) - + + +
Piplartine (μM) 0 0 5 10
60
45
35
p-P38: 43 kDa
60
45
35
P38: 43 kDa

Figure.S2

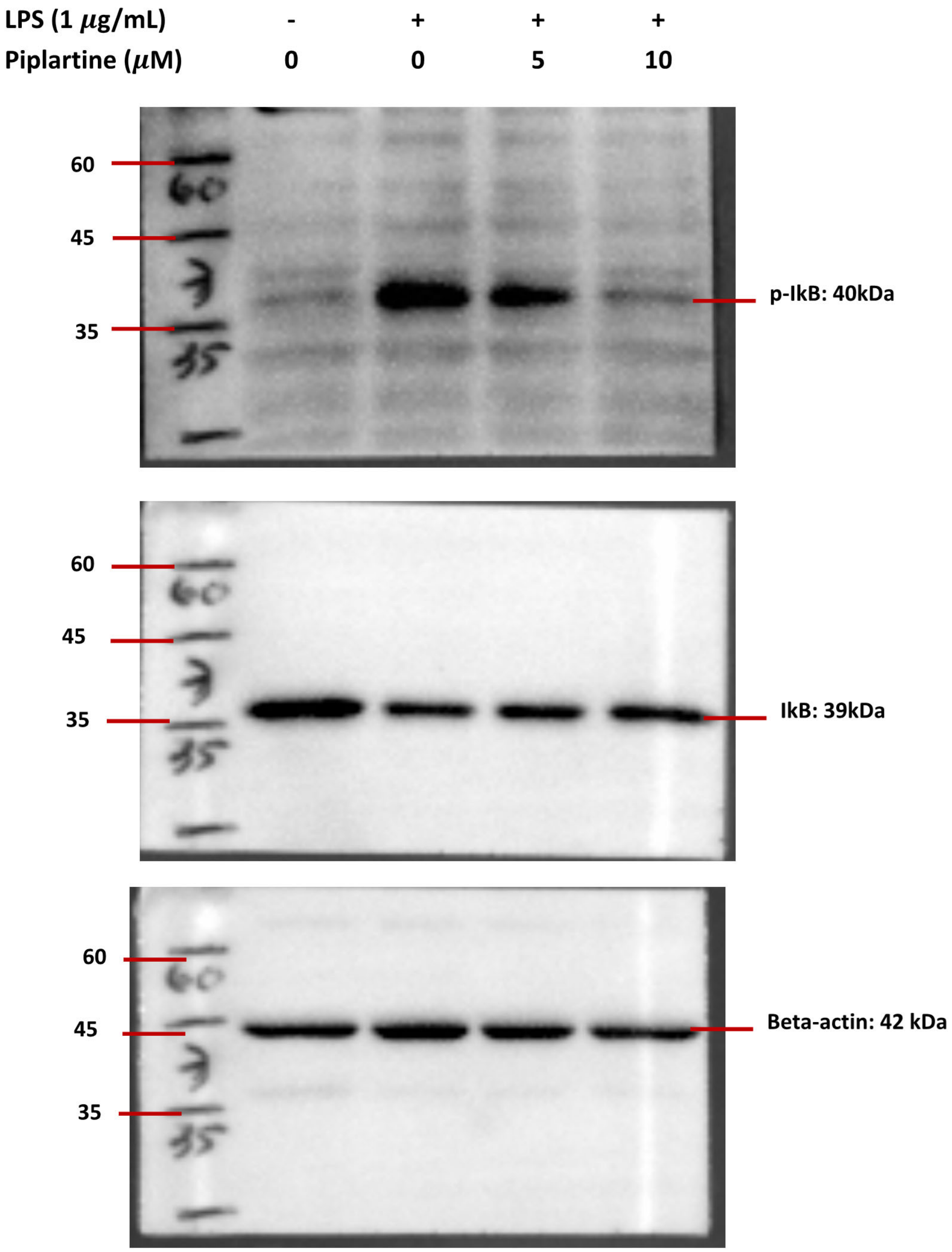
LPS (1 μg/mL) - + + +
Piplartine (μM) 0 0 5 10
60
45
35
p-IkB: 40kDa
60
45
35
IkB: 39kDa
60
45
35
Beta-actin: 42 kDa

Figure.S2

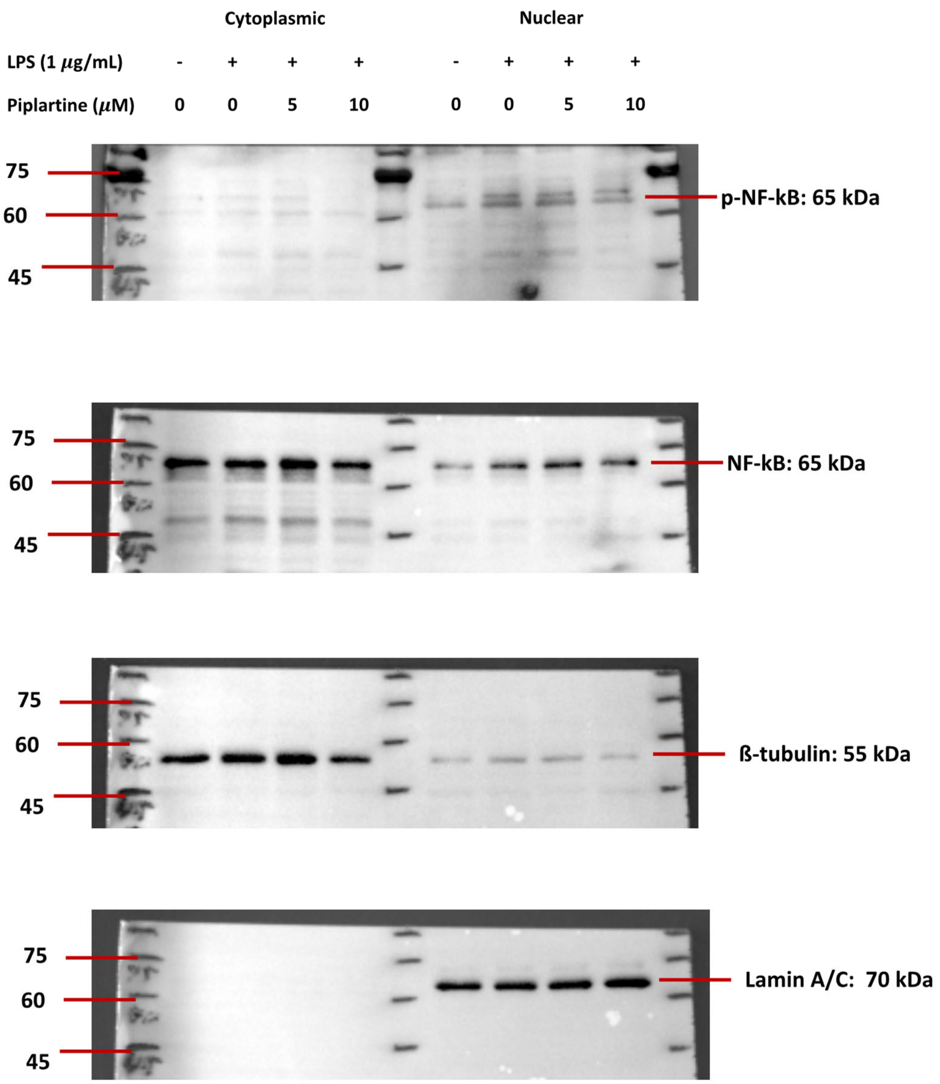

Cytoplasmic
Nuclear
LPS (1 μg/mL) - + + + - + + +
Piplartine (μM) 0 0 5 10 0 0 5 10
75
60
45
p-NF-kB: 65 kDa
75
60
45
NF-kB: 65 kDa
75
60
45
ß-tubulin: 55 kDa
75
60
45
Lamin A/C: 70 kDa

Figure.S3

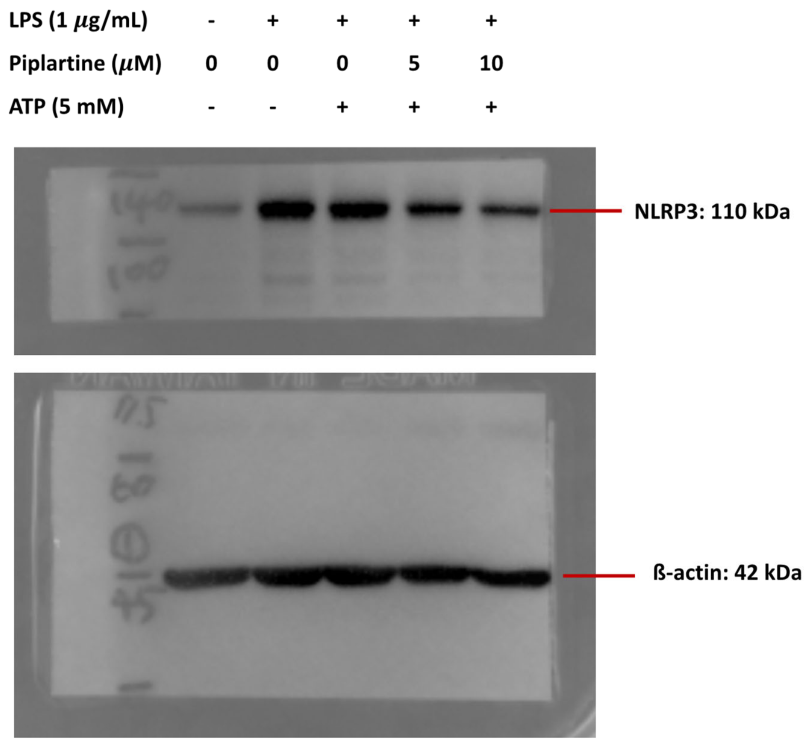
LPS (1 µg/mL) - + + + +
Piplartine (µM) 0 0 0 5 10
ATP (5 mM) - - + + +
NLRP3: 110 kDa
ß-actin: 42 kDa

Figure.S3

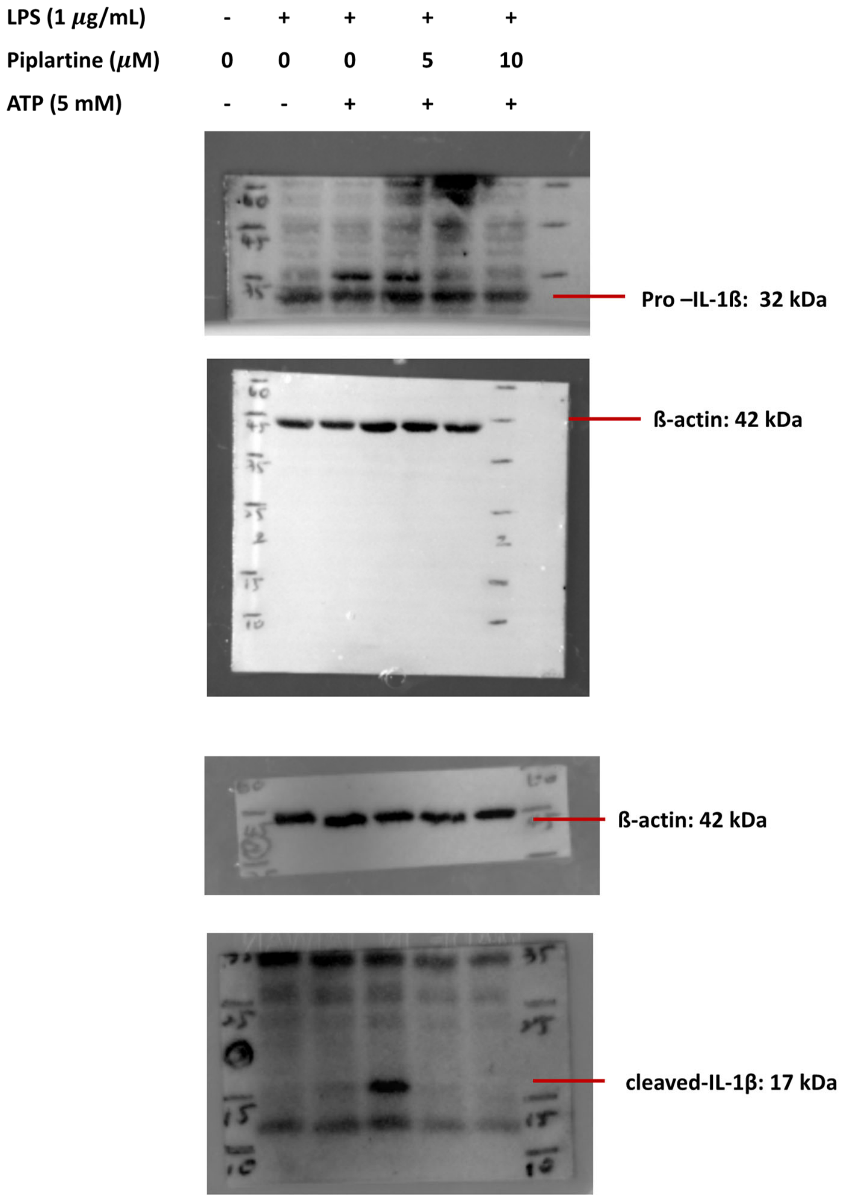
LPS (1 μg/mL) - + + + +
Piplartine (μM) 0 0 0 5 10
ATP (5 mM) - - + + +
Pro –IL-1ß: 32 kDa
ß-actin: 42 kDa
ß-actin: 42 kDa
cleaved-IL-1β: 17 kDa

Figure.S3

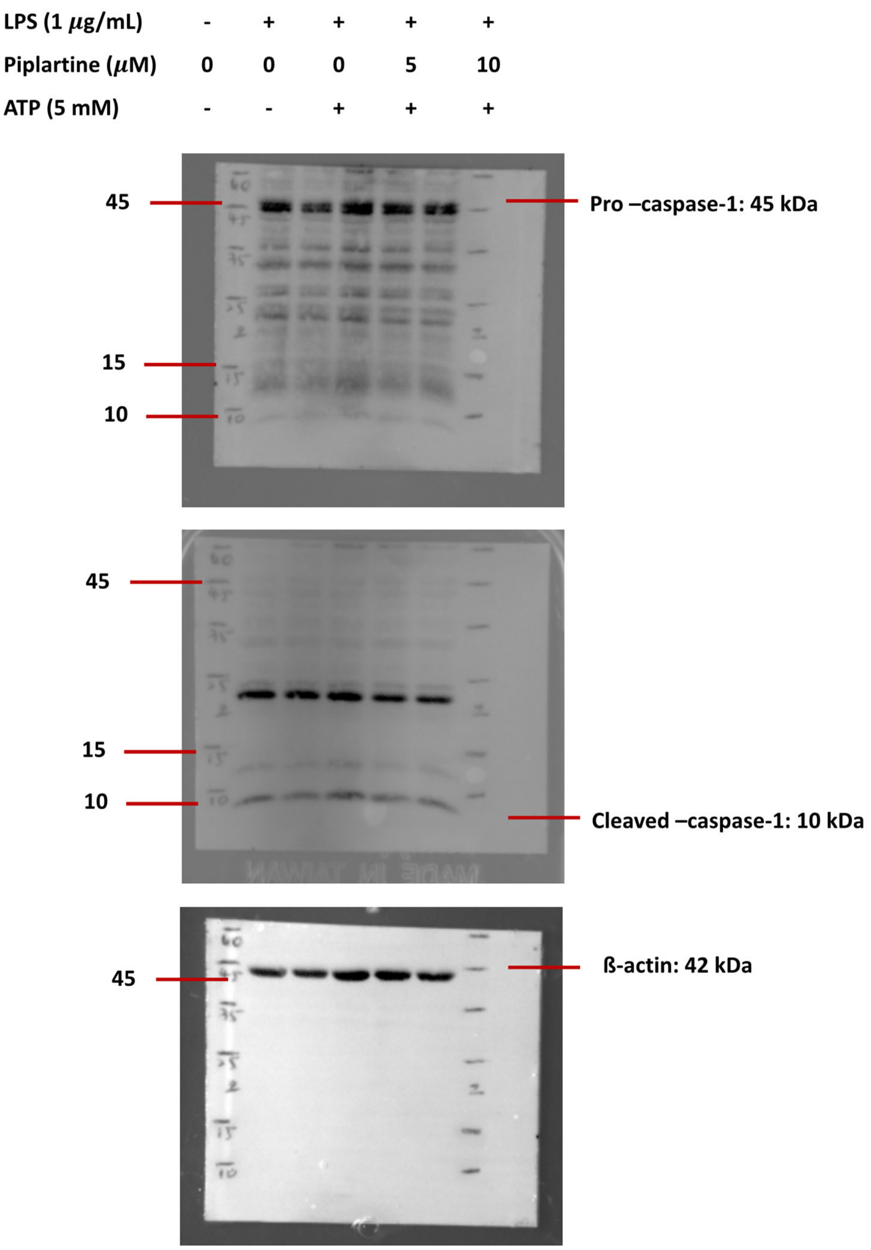

LPS (1 μg/mL) - + + + +
Piplartine (μM) 0 0 0 5 10
ATP (5 mM) - - + + +
45
15
10
Pro –caspase-1: 45 kDa
45
15
10
Cleaved –caspase-1: 10 kDa
45
ß-actin: 42 kDa

Figure.S3

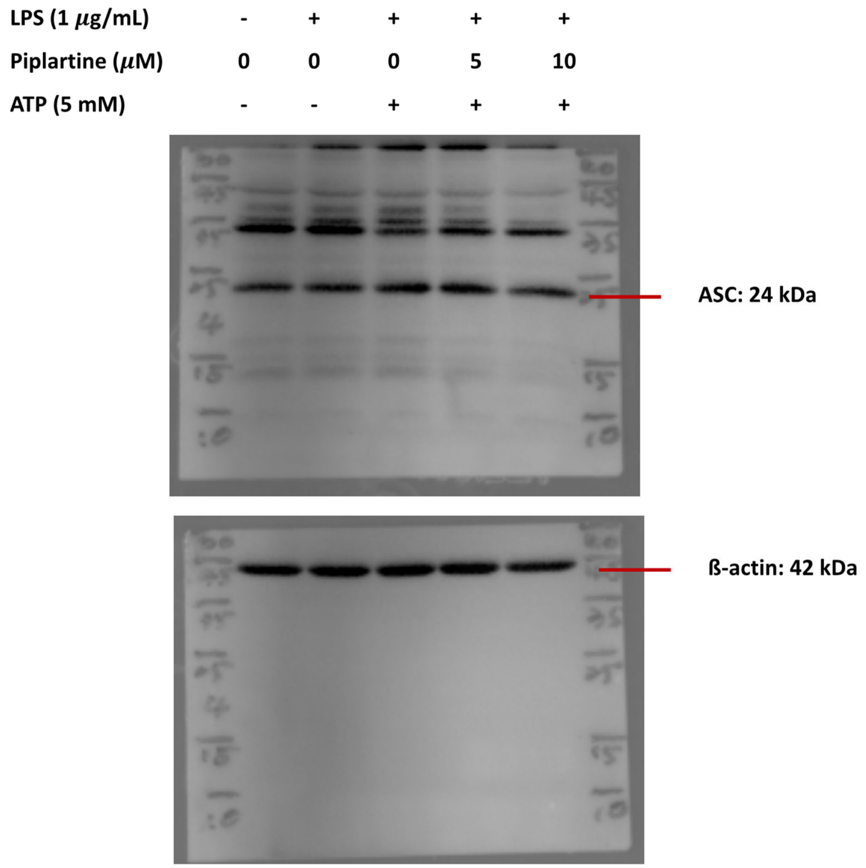
LPS (1 μg/mL) - + + + +
Piplartine (μM) 0 0 0 5 10
ATP (5 mM) - - + + +
ASC: 24 kDa
ß-actin: 42 kDa
